# The Burden and Treatment of Chronic Constipation Among US Nursing Home Residents

**DOI:** 10.1016/j.jamda.2023.05.006

**Published:** 2023-06-09

**Authors:** Tingting Zhang, Andrew R. Zullo, Hannah O. James, Yoojin Lee, Douglas C.A. Taylor, Lori A. Daiello

**Affiliations:** a Department of Health Services, Policy, and Practice, Brown University School of Public Health, Providence, RI, USA; b Department of Epidemiology, Brown University School of Public Health, Providence, RI, USA; c Center of Innovation in Long-Term Services and Supports, Providence Veterans Affairs Medical Center, Providence, RI, USA; d Department of Pharmacy, Lifespan—Rhode Island Hospital, Providence, RI, USA; e Ironwood Pharmaceuticals, Boston, MA, USA

**Keywords:** Constipation, laxatives, nursing homes, electronic health records, Medicare

## Abstract

**Objective:**

To evaluate the burden of chronic constipation (CC) and the use of drugs to treat constipation (DTC) in 2 complementary data sources.

**Design:**

Retrospective cohort study.

**Setting and Participants:**

US nursing home residents aged ≥65 years with CC.

**Methods:**

We conducted 2 retrospective cohort studies in parallel using (1) 2016 electronic health record (EHR) data from 126 nursing homes and (2) 2014–2016 Medicare claims, each linked with the Minimum Data Set (MDS). CC was defined as (1) the MDS constipation indicator and/or (2) chronic DTC use. We described the prevalence and incidence rate of CC and the use of DTC.

**Results:**

In the EHR cohort, we identified 25,739 residents (71.8%) with CC during 2016. Among residents with prevalent CC, 37% received a DTC, with an average duration of use of 19 days per resident-month during follow-up. The most frequently prescribed DTC classes included osmotic (22.6%), stimulant (20.9%), and emollient (17.9%) laxatives. In the Medicare cohort, a total of 245,578 residents (37.5%) had CC. Among residents with prevalent CC, 59% received a DTC and slightly more than half (55%) were prescribed an osmotic laxative. Duration of use was shorter (10 days per resident-month) in the Medicare (vs EHR) cohort.

**Conclusions and Implications:**

The burden of CC is high among nursing home residents. The differences in the estimates between the EHR and Medicare data confirm the importance of using secondary data sources that include over-the-counter drugs and other treatments unobservable in Medicare Part D claims to assess the burden of CC and DTC use in this population.

Chronic constipation (CC) is associated with significant adverse consequences and meaningful decrements in quality of life (QoL)^1–3^ among older adults. In the community, more than 20% of US adults aged ≥65 years report CC; however, reports vary according to the population(s) under study and the operational definition of the condition.^4^ Nursing home (NH) residents are at particularly high risk for CC because of a multiplicity of contributing factors that include advanced age, medical complexity, polypharmacy, insufficient fluid intake, impaired mobility, and reduced levels of physical activity.^3^ Not surprisingly, the prevalence is substantial in these settings, where CC is estimated to affect up to 80% of long-stay NH residents.^5–9^

Given the rapidly evolving landscape of drug development and treatment guidelines for other highly prevalent chronic conditions (eg, hypertension, diabetes), optimal management of CC may be viewed as a lower priority. This perspective is expected given that the most frequently prescribed treatments for CC are over-the-counter (OTC) medications, including some that have been in use for >50 years.^10^ However, if not adequately controlled, CC can lead to potentially life-threatening complications, including fecal impaction and bowel obstruction.^6,11^ Less recognized is the possible impact of CC on other comorbid conditions, for example, cardiovascular disease, where events such as syncope, atrial fibrillation, and exacerbation of heart failure following excessive straining have been described.^12^

A better understanding of the epidemiology of CC and its management is essential to understanding the burden of CC and the care needs of NH residents. Some of the most recent studies conducted in US NHs were completed >2 decades ago.^13^ Notably, this research predates important developments in the field, such as the availability of newer prescription agents that treat CC through novel mechanisms^11^ and rising interest in probiotics.^14^

Nonetheless, an important caveat must be considered when designing such a study; with a few exceptions (eg, polyethylene glycol), the OTC laxatives that are the mainstay of CC treatment are not covered under the Medicare Part D program and are thus unobservable in Part D administrative claims. Electronic health records (EHRs) contain comprehensive clinical documentation and may be an alternate resource for medication data not found in administrative claims.

Our primary objective was to evaluate the burden of CC and its treatment among long-stay NH residents in the United States in 2 complementary data sources. The secondary objective was to estimate hospitalization costs associated with constipation-related conditions.

## Methods

### Study Design and Data Sources

We conducted 2 retrospective cohort studies using separate, but complementary, data sources. The first used 2016 EHRs from a nationally representative sample of 126 NHs operated by a single US provider of post-acute and long-term care (EHR cohort). The data set contained physician orders, nursing notes, and medication administration records for a 1-year period (January 1, 2016, to December 31, 2016). Medication records included prescription drug and OTC drug names, dose, and quantity dispensed, as well as documentation of dates and times of administration.

The second study (Medicare cohort) used national Medicare fee-for-service Parts A and D claims (January 1, 2014, to December 31, 2016) and the 100% national Medicare Master Beneficiary Summary File (MBSF).

Each data source was linked to the Minimum Data Set (MDS, version 3.0). The MDS is a federally mandated assessment performed at admission, quarterly thereafter, and upon significant change in status and discharge from the NH.^15^ Key variable domains in each data source are summarized in Supplementary Table 1.

The study protocol was approved by the Brown University institutional review board.

### Study Population

The study population was long-stay NH residents aged 65 years or older with CC. Because NH admission and discharge dates were not available for all residents in the EHR data set, the standard CMS algorithm for selecting long-stay NH populations could not be applied.^16^ Therefore, we employed an alternate approach, in which long-stay qualification was met when the interval between dates of consecutive MDS assessments, or between an MDS assessment and medication administration date, was at least 100 days. For the Medicare cohort, *long stay* was defined as residence in the same facility for at least 100 days (with no more than 10 consecutive days outside the facility).^16^ The first day of long-stay qualification was designated as the index date. Demographics and clinical characteristics were identified during the baseline period, defined as the 100-day period preceding the index date (Supplementary Figure 1).

Those lacking complete data, or who had disenrolled from Medicare Parts A or D, or enrolled in a Medicare Advantage plan (Medicare cohort) during the study period were excluded. Residents with a primary inflammatory or organic gastrointestinal diagnosis (eg, colorectal cancers, inflammatory bowel disease, ulcerative colitis, or Crohn disease) prior to cohort entry were excluded.

### Measures

#### Definition of chronic constipation

Clinically, constipation is typically defined as fewer than 3 bowel movements weekly and/or stools that are hard or difficult to pass. The Rome IV criteria for functional bowel disorders (ie, chronic constipation) are employed by gastroenterology and for research but their complexity limits routine clinical use.^17^ Identifying CC in secondary data is challenging; although laxatives and treatments are often prescribed, a diagnosis of constipation or CC is often undocumented.^18^ Furthermore, there are no validated algorithms for identifying constipation in Medicare claims or EHR data. For this study, we used a composite definition, in which CC was coded if one or both criteria were met: (1) the MDS indicator for constipation was checked (item H0600) and/or (2) chronic DTC use.

#### Chronic DTC Use

For the EHR cohort, data from administration records defined chronic DTC use as (1) a scheduled DTC routinely administered for ≥60 days, or (2) DTCs prescribed pro re nata (PRN) or “when needed” administered 1 or more times per week for ≥60 days. Duration of treatment was calculated as the difference (in days) between the first and last dates of routine administration.

In the Medicare cohort, chronic DTC use was defined in Medicare Part D claims by sequential dispensings, when the total quantity of medication supplied was ≥60 days. We evaluated the use of all prescription and nonprescription laxatives and other drugs indicated for constipation treatment during baseline and follow-up (Supplementary Table 2).

#### Outcomes

Outcomes included the frequency of CC and utilization of DTCs. In the Medicare cohort with CC, we also estimated rates of hospitalizations in which potential constipation-related conditions (CRCs) were coded, the average cost of hospitalization, and DTC use per resident month during follow-up. Health services utilization and cost information could not be estimated for the EHR cohort because of data use agreement restrictions that prohibited linkage with other data sources.

*International Classification of Diseases, Ninth and Tenth Revisions (ICD-9 and ICD-10)* diagnosis and procedure codes in the primary or any secondary position in claims diagnosis fields identified hospitalizations that included 1 or more CRCs. Selection of qualifying codes was informed by clinical experience and review of CRCs previously used in health services research (Supplementary Table 4). Hospitalization costs were based on the Medicare reimbursement dollar amount documented in Part A claims. Drug expenditures were estimated from cost information in Medicare Part D claims.

#### Follow-up

Follow-up began on the first day of long-stay qualification (index date) and continued until the earliest occurrence of December 31, 2016, date of death, or (1) the last MDS assessment date or drug order (EHR cohort), or (2) until Medicare disenrollment or NH discharge (Medicare cohort).

#### Resident Characteristics

We described residents with CC by demographic characteristics, physical and cognitive function, mortality risk score, and comorbidities at baseline. Physical functioning was characterized using the 28-point MDS Activities of Daily Living (ADL) Long Form Scale.^19^ We used the Cognitive Function Scale (CFS) to measure cognitive function, which categorizes severity of cognitive impairment from 1 (no or minimal) to 4 (severe).^20^ Mortality risk was estimated by the Changes in Health, End-stage disease Symptoms and Signs (CHESS) score, a validated measure in NH populations, ranging from 0 (stable) to 5 (high health instability).^21^ Active health conditions and common comorbidities were extracted from the MDS.

#### Statistical Analysis

Prevalence was calculated by summing the number of residents classified with CC at baseline and/or during follow-up, divided by the population at risk for CC (ie, number of residents who did not meet criteria for CC during baseline). Incidence rates were calculated by dividing the number of residents who met CC criteria only during follow-up by the total number of resident-days for the population at risk for CC. To quantify the precision of the estimates, 95% CIs were calculated for prevalence calculations and incidence rates. DTC utilization was calculated as (1) the proportion of residents with constipation who received a DTC, by class and individual product; and (2) days of therapy for each drug class per resident month among prevalent cases.

SAS software, version 9.3 (SAS Institute, Cary, NC), and Statistical Package for the Social Sciences (SPSS), version 24.0 (IBM Corp), were utilized for the analyses.

## Results

### EHR Cohort

After applying selection criteria, the EHR cohort included 35,858 long-stay residents (Supplementary Table 3). The composite definition classified 25,739 or 71.8% (95% CI 71.3–74.2) with prevalent CC. On average, residents with CC were >80 years [mean age, 81.3 (SD 8.7) years] and female (64%); 81% were White and 13% were Black. The most common comorbidities included hypertension (80%), diabetes (36%), and gastrointestinal conditions (36%) (Table 1).

There were 15,352 residents without CC at baseline. Of these, 5233 residents (34.1%) were identified with incident CC during mean follow-up time of 232 (SD) 127 days [incidence rate, 0.64 (95% CI 0.63–0.66)] per 1000 resident-days.

### EHR cohort: DTC utilization

Among residents with CC, 86.0% received a DTC during the observation period; of those, 65% received a DTC at baseline and 36.6% received a DTC during follow-up. The most frequently prescribed classes included osmotic (22.6%), stimulant (20.9%), and emollient (17.9%) laxatives. The average duration of use was 19 days per resident-month during follow-up; among the DTC classes, emollient laxatives had the longest duration of use (17 days of therapy per resident-month) (Table 2).

The most frequently prescribed individual products included sodium/calcium docusate, sennosides, polyethylene glycol (PEG), and milk of magnesia. Few residents received one of the prescription medications with an FDA indication for CC; linaclotide was prescribed for 0.6% (n = 149) and lubiprostone for 0.3% (n = 81) of residents with CC. Neither plecanatide nor prucalopride were prescribed during the study.

### Medicare Cohort

A total of 655,543 long-stay residents were included in the Medicare cohort. The composite CC definition classified 245,578 or 37.5% (95% CI 37.3–37.6) with prevalent CC. The mean age was 82.8 (SD) 8.9 years and 73% were female; 83% were White and 13% Black. The most common comorbidities included hypertension (83.5%), dementia / Alzheimer’s disease and related dementias (66.5%), and diabetes (36.5%).

Among 566,543 residents without CC at baseline, a total of 156,485 residents (27.6%) were identified with incident CC, with mean follow-up time of 566 (SD) 214 days (incidence rate, 0.47, 95% CI 0.47–0.48) per 1000 resident-days.

### Medicare cohort: DTC utilization

Among the prevalent cohort with CC, 62.5% received a DTC during the study; 36% of those received a DTC at baseline, and 58.5% received a DTC during follow-up. The average duration of use during follow-up was 10 days per resident-month.

The most frequently prescribed class was osmotic laxatives (55.7%), and within the osmotic laxative class, PEG was used most frequently (40.5%). Utilization of newer prescription medications for CC was modest, but higher than in the EHR cohort; linaclotide, lubiprostone, and either prucalopride or plecanatide were prescribed for 3.6%, 2.6%, and 0.1% of residents with prevalent CC, respectively (Table 2).

### Medicare cohort: hospitalizations and costs

A total of 20,295 residents had a hospitalization with a CRC during follow-up (8.1%). The estimated average cost per resident-month was $43.40, representing 12.2% of the average cost for all-cause hospitalizations ($355.50 per resident-month). The average combined total cost of DTC and hospitalizations with CRCs per resident-month was $58.60, which accounted for 16.5% of the average cost of all-cause hospitalizations per resident-month of $355.50 (Table 3).

## Discussion

This is the first study to demonstrate substantial differences between the prevalence of CC observed in an EHR data set (71.8%) vs Medicare claims (37.5%). Despite the notable variation in these estimates, both are within the range of values reported in previous studies of other NH populations where the prevalence of constipation was 50% in a US cohort,^22^ 23.4% and 71.5% in 2 Norwegian cohorts,^5,6^ and 48% in an Italian cohort.^23^ A novel methodologic aspect of the current study is the use of complementary data sources that differ in the level of detail for OTC laxative utilization. The Medicare Part D program does not cover nonprescription medications or treatments, with few exceptions.^24^ This characteristic prohibits the use of Part D data for studying CC treatment, which primarily consists of OTC oral laxatives, stool softeners, and enemas. By contrast, EHR clinical documentation contains “real-time” treatment information, which includes utilization of all prescription and OTC laxatives, as well as the frequency of PRN administration. Without these data, an accurate description of CC epidemiology and patterns of OTC laxative prescribing in NHs cannot be determined.

OTC laxatives comprised most of the pharmacologic treatment of CC. Over 80% of the EHR cohort and nearly two-thirds of long-stay NH residents in the Medicare cohort received a DTC during the study. The mean duration of therapy was 19 days per resident-month in the EHR cohort (vs 10 days per resident-month in the Medicare cohort), suggesting chronicity of laxative therapy. Chronic OTC laxative use is common in adults with CC and generally regarded as safe, but there is scant evidence of long-term effectiveness and safety in NH populations, where polypharmacy and multimorbidity heighten the risk of adverse effects. Except for several studies of PEG,^25^ few laxative trials have enrolled adequate numbers of older adult subjects, utilized active comparators, or evaluated treatment outcomes beyond 3 months.^26^ In a recent systematic review of NH clinical trials, Alsalimy et al identified only 7 randomized controlled OTC laxative trials and found that the duration of treatment ranged from 14 days to 8 weeks.^27^

Similarly, there is little published evidence from trials conducted in older adult populations to inform appropriate use of the newer prescription medications indicated for CC. Evaluation of treatment options for CC by the American College of Gastroenterology^28^ and others^29,30^ indicate use of an intestinal secretagogue^31^ (linaclotide, lubiprostone, or plecanatide) if first-line interventions (eg, lifestyle and dietary modifications, eliminating medications that cause or exacerbate constipation, use of OTC laxatives) are ineffective. We observed low utilization of newer prescription treatments for CC in both cohorts. This observation may be related to higher cost of newer therapies, compared with OTC laxatives, and insufficient data for safety and effectiveness in older adults.

The economic burden of health care resource utilization is considerable and well-documented in population-based CC studies^32,33^; comparable data for NH populations is lacking. Among long-stay NH residents with CC in the Medicare cohort, we estimated that expenditures for hospitalizations with CRCs and DTC costs accounted for 16.5% of the average per-resident-month cost for all-cause hospitalizations during follow-up. Although preliminary, these results suggest that CC-related sequelae (which may be preventable, in part) are a nontrivial component of the cost of all-cause hospitalizations. The economic burden of CC is also present at the NH level. In 2002, Frank and colleagues^13^ conducted a time study of constipation care and bowel protocol tasks at 2 large NHs. Nursing administration of oral laxatives was the most time-consuming (and costly) aspect of care; the annual inflation-adjusted labor costs were estimated at $2597 per resident with constipation.

The humanistic burden of CC^33^ is broadly defined by its effects on quality of life (QoL). Studies of healthy older adults with CC link symptoms of flatulence, bloating, abdominal pain, rectal pain, and sudden urge to defecate with emotional distress and frequent disruption of daily routines.^34^ The effects of CC on emotional well-being and QoL of NH residents are not as well studied but are of particular concern for residents unable to communicate their experiences because of cognitive impairment or other limitations. Negative impacts of CC on QoL domains^35^ and other outcomes have been documented, for example, onset or exacerbation of fecal incontinence,^36^ anorexia,^37^ agitated behaviors in dementia,^38^ increased utilization of benzodiazepines,^39,40^ and reduced participation in activities.^40^

Our results must be considered within the context of potential limitations. By focusing on CC and emphasizing routine DTC use as a criterion in our case definitions, we may have underestimated prevalence by failing to capture undertreated constipation. However, the impact of assessing only routine DTC use is unclear, as the prevalence of constipation found in the EHR analyses is not dissimilar from results reported in other studies of constipation and its treatment in NHs. Second, information for residents enrolled in Medicare Advantage plans was unavailable, so the extent to which our findings are applicable to this sub-population is unknown. Third, the study was not designed to determine causal relationships between CC and hospitalizations arising from constipation-related sequelae; therefore, the health care utilization and cost estimates are preliminary and warrant future research. Fourth, the EHR data were from 1 for-profit, post-acute care NH corporation, which may limit the generalizability of our findings to privately owned NHs or to those managed by other organizations. Other caveats related to the EHR data structure include (1) 1 year of available data, which restricted duration of follow-up (8 months, EHR cohort vs Medicare cohort, 19 months) and limited interpretation of observed differences (eg, CC incidence rate) between cohorts and (2) without linkage to Medicare data, supplemental information (eg, payor type, health services utilization) was inaccessible and the total duration of NH residence could not be calculated. Of recent note, investigators considering the use of EHR data may avoid these issues by utilizing the LTC Data Cooperative, a national repository of multiyear EHR data sets linked with Medicare claims, established in 2022.^41^

## Conclusions and Implications

CC is highly prevalent in NH populations and significantly impacts QoL and health care resources. This study provides an updated description of CC epidemiology and laxative utilization in US nursing homes. We anticipate that these findings will inform the efforts of researchers seeking to address knowledge gaps regarding pharmacologic treatments for CC and other interventions aimed at improving its management.

## Supplementary Material

1

## Figures and Tables

**Table 1:** Characteristics of Long-Stay NH Residents With Prevalent Chronic Constipation

	EHR Cohort (n = 25,739)	Medicare Cohort (n = 245,578)
Prevalence of CC, %	71.8	37.5
Demographics		
Age, y, mean (SD)	81.3 (8.7)	82.8 (8.8)
Women, n (%)	16,506 (64.1)	180,375 (73.4)
Race, n (%)		
White	20,885 (81.1)	203,560 (82.9)
Black	3379 (13.1)	30,602 (12.5)
Others	1064 (4.1)	10,887 (4.4)
Unknown	411 (1.6)	529 (0.2)
Geriatric syndromes, n (%)		
CHESS score		
0 (most stable)	12,414 (48.2)	136,279 (55.5)
1	8050 (31.3)	73,104 (29.8)
2–5 (moderate to severe health instability)	5264 (20.5)	35,691 (14.5)
Cognitive Function Scale (CFS) score		
No or minimal impairment (CFS = 1)	14,059 (54.6)	84,071 (34.2)
Mild or moderate impairment (CFS = 2 or 3)	10,563 (41.0)	139,611 (56.8)
Severe impairment (CFS = 4)	1105 (4.3)	21,700 (8.8)
ADL score		
Mild to moderate impairment (ADL<21)	21,390 (83.1)	185,203 (75.4)
Severe impairment (ADL≥21)	4349 (16.9)	60,326 (24.6)
Daily pain	1201 (4.7)	23,993 (9.8)
Selected comorbidities, n (%)		
Cardiovascular		
Congestive heart failure	6287 (24.4)	71,326 (29.0)
Hypertension	20,688 (80.4)	205,115 (83.5)
Neurologic		
Dementia/ADRD	7717 (30.0)	163,364 (66.5)
Cerebrovascular accident, transient ischemic attack, stroke	1912 (7.4)	54,517 (22.2)
Multiple sclerosis	209 (0.8)	2915 (1.2)
Parkinson’s disease	1173 (4.6)	22,609 (9.2)
Metabolic		
Diabetes	9156 (35.6)	89,705 (36.5)
Thyroid disorder	5643 (21.9)	65,721 (26.8)
Genitourinary		
Renal insufficiency, acute renal failure, or end-stage renal disease	5306 (20.6)	36,733 (15.0)
Cancer	2960 (11.5)	14,742 (6.0)
Gastrointestinal	9348 (36.3)	68,734 (28.0)
Bowel and bladder function		
Bladder incontinence	10,970 (42.6)	189,463 (77.1)
Bowel incontinence	10,525 (40.9)	182,293 (74.2)

ADL, activities of daily living; ADRD, Alzheimer’s disease and related dementias; CHESS, Changes in Health, End-stage disease and Symptoms and Signs.

**Table 2:** DTC Utilization Among Long-Stay NH Residents With Prevalent Chronic Constipation

	EHR Cohort (n = 25,739)	Medicare Cohort (n = 245,578)
Laxative class, n (%)		
Bulk-forming	347 (1.3)	79 (0.03)
Emollient	4599 (17.9)	401 (0.2)
Osmotic	5807 (22.6)	136,709 (55.7)
Stimulant	5370 (20.9)	220 (0.1)
DTC utilization, n (%)		
Bulk-forming		
Bulk-forming powders	455 (1.8)	—
Bulk-forming tablets	207 (0.8)	—
Emollient		
Sodium/calcium docusate	4512 (17.5)	397 (0.2)
Osmotic		
Polyethylene glycol	3782 (14.7)	99,489 (40.5)
Milk of magnesia	2416 (9.4)	N/A
Lactulose	796 (3.1)	48,328 (19.7)
Stimulant		
Sennosides	4322 (16.8)	142 (0.1)
Prescription medications for constipation		
Linaclotide	149 (0.6)	8907 (3.6)
Lubiprostone	81 (0.3)	6355 (2.6)
Other*	N/A	160 (0.1)

N/A, no utilization.

Blank cells indicate results for fewer than 11 residents, which are not reportable under CMS Data Use Agreements.

* Other: plecanatide and prucalopride.

**Table 3:** Estimated Costs Associated With Hospitalizations That Included Potential Constipation-Related Conditions and Drugs to Treat Constipation (DTCs) During Follow-up Among Residents With Chronic Constipation (Medicare Cohort)

	Total Costs ($)	Cost ($) per Resident-Month
DTC	73,601,109	15.20
Hospitalizations with constipation-related conditions	210,096,717	43.40
DTC + hospitalizations with constipation-related conditions	283,697,826	58.60
All-cause hospitalizations	1,722,027,478	355.50

Costs of hospitalizations based on reimbursement dollar amount documented in Medicare Part A claims. Cost of DTC was based on gross cost for each drug product in Medicare Part D claims.
