# Supplementary material for: The Burden and Treatment of Chronic Constipation Among US Nursing Home Residents
Source: J Am Med Dir Assoc. Author manuscript; Available in PMC 2023 Nov 13. (PMC10642798; doi:10.1016/j.jamda.2023.05.006)
Supplement: 1 [file NIHMS1938998-supplement-1.pdf]

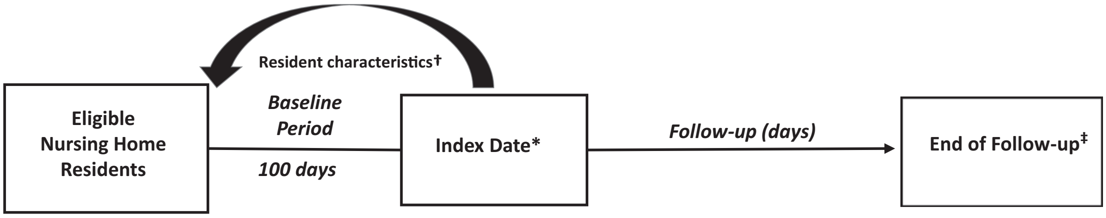

Supplementary Fig. 1. Study design diagram. *The index date is the first day of long-stay qualification, defined as residence in the same nursing home facility for ≥100 days, with no more than 10 days outside the facility (Medicare cohort), or at the first observation of an interval of ≥100 days between either the dates of consecutive MDS assessments, or between an MDS assessment and medication administration date (EHR cohort). †Demographic and clinical characteristics are extracted from the Minimum Data Set (MDS) assessment closest to and preceding the index date. ‡Date of the earliest event of December 31, 2016, death, or (1) Medicare disenrollment or NH discharge (Medicare cohort) or (2) date of the last MDS assessment or medication order in the dataset (EHR cohort).

Supplementary Table 1
Summary of Key Variable Domains for Each Study Cohort

Specific Measure	Medicare Cohort	EHR Cohort
Medicare enrollment	Medicare Beneficiary Summary File (Parts A and D enrollment data)	Not available
Demographics	Medicare Beneficiary Summary File	MDS data
Death dates	Medicare Beneficiary Summary File	Not available
NH admission and discharge dates	Residential History File	Not available
Use of drugs to treat constipation	Medicare Part D prescription drug claims	Medication administration records (including prescription drugs, over-the-counter products, administration of PRN drugs)
Hospitalization events	Medicare Part A hospital admission and discharge dates, diagnoses	Not available
Costs	Medicare Parts A and D reimbursement costs	Not available
Duration of NH stay	NH admission and discharge dates in the Residential History File	Assessment dates in MDS data
Clinical characteristics	The MDS data linked to each data source	

Definitions: Nursing home, (NH); Electronic health record, (EHR); Minimum Data Set, (MDS); Pro re nata, (PRN), administration of drugs or treatment as needed.

Supplementary Table 2
Medications for Constipation Included in the Study

Osmotic	Bulk-forming
Polyethylene glycol (PEG)	Psyllium
Lactulose	Calcium polycarbophil
Sodium phosphate	Methylcellulose
Magnesium hydroxide	Cellulose
Magnesium citrate	Wheat dextrin
Sorbitol	Corn dextrin powder
Stimulant	Emollient
Bisacodyl	Docusate sodium
Sennosides	Mineral oil
Sennosides docusate sodium	Glycerin suppository
Phenolphthalein	Other
Cascara sagrada	Lubiprostone
Castor oil	Linaclotide
	Plecanatide
	Prucalopride

Supplementary Table 3
Medicare and EHR Cohort Selection

Exclusion Criteria	Medicare Cohort	EHR Cohort
	Long-stay NH residents with 1 or more MDS assessments	
	n = 920,713	n = 44,477
Aged <65 y	9489	8024
Not enrolled in Medicare Part A	18,719	—
Not enrolled in Medicare Part D	58,674	—
Enrolled in a Medicare Advantage program	172,598	—
No prescription drug claims for any medication, despite enrollment in Medicare Part D	2977	—
MDS documentation of colorectal cancer, inflammatory bowel disease, ulcerative colitis, or Cohn disease prior to cohort entry	2713	595
Number of residents included in the final analytic sample	655,543	35,858

NH, nursing home; EHR, Electronic health record; MDS, Minimum Data Set.

Supplementary Table 4
Diagnosis Codes Considered in Analyses of Costs Associated With Hospitalizations That Included Constipation-Related Conditions

ICD-9 Codes	*ICD-10* Codes
455.xx Hemorrhoids	K64.0 First degree hemorrhoids
	K64.1 Second degree hemorrhoids
	K64.2 Third degree hemorrhoids
	K64.3 Fourth degree hemorrhoids
	K64.4 Residual hemorrhoidal skin tags
	K64.5 Perianal venous thrombosis
	K64.8 Other hemorrhoids
	K64.9 Unspecified hemorrhoids
560.10 Paralytic ileus	K56 Paralytic ileus and intestinal obstruction without hernia
	K56.7 ileus, unspecified
560.32 Fecal impaction	K56.41 Fecal impaction
560.9 Intestinal obstruction, unspecified	K56.69 Intestinal obstruction, other
578.1 Blood in stool	K92.1 Melena
783.21 Loss of weight	R63.4 Abnormal weight loss
787.01 Nausea with vomiting	R11.2 Nausea with vomiting, unspecified
787.02 Nausea alone	R11.0 Nausea
	R11.11 Nausea without vomiting
787.3 Flatulence eructation/gas	R14.0 Abdominal distension (gaseous)
	R14.1 Gas pain
	R14.2 Eructation
	R14.3 Flatulence
787.91 Diarrhea	R19.7 Diarrhea, unspecified
	K52.89 Other specified noninfective gastroenteritis and colitis
787.99 Other digestive symptoms	R19.4 Change in bowel habit
789.00 Abdominal pain unspecified site	R10.9 Unspecified abdominal pain
789.01 Abdominal pain right upper quadrant	R10.11 Right upper quadrant pain
789.04 Abdominal pain left lower quadrant	R10.32 Left lower quadrant pain
789.06 Abdominal pain, epigastric	R10.13 Epigastric pain

ICD, International Classification of Diseases.
Costs were estimated using reimbursement dollar amounts in Medicare Part A claims (Medicare cohort).
